# ANP32A Knockdown Attenuates the Malignant Biological Behavior of Colorectal Cancer Cells by Suppressing Epithelial-mesenchymal Transition and ERK Activation

**DOI:** 10.7150/jca.84687

**Published:** 2023-09-04

**Authors:** Xiaojuan Li, Xumei Li, Guoxiang Liu, Luwei Zhou, Yisa Liu, Tong Dou, Xu Chen, Juan Wang

**Affiliations:** 1 Department of Pharmacy, Guilin Medical University, Guilin, Guangxi Zhuang Autonomous Region 541199, China.; 2 Guangxi Key Laboratory of Molecular Medicine in Liver Injury and Repair, The Affiliated Hospital of Guilin Medical University, Guilin, Guangxi Zhuang Autonomous Region 541001, China.; 3 Guangxi Health Commission Key Laboratory of Basic Research in Sphingolipid Metabolism Related Diseases, The Affiliated Hospital of Guilin Medical University, Guilin, Guangxi Zhuang Autonomous Region 541001, China.; 4 Faculty of Basic Medicine, Guilin Medical University, Guilin, Guangxi Zhuang Autonomous Region 541004, China.; 5 School of Pharmacy, Macau University of Science and Technology, Avenida Wai Long, Taipa, Macau, China.

**Keywords:** Acidic leucine rich nuclear phosphoprotein-32A, metastasis, epithelial-mesenchymal transition, β-catenin, ERK, colorectal cancer

## Abstract

Acidic leucine rich nuclear phosphoprotein-32A (ANP32A) protein has a variety of functions, such as regulating cell differentiation, influencing cell apoptosis and cell cycle progression. Our previous study demonstrated that high expression of ANP32A was found in the tumor tissues of colorectal cancer (CRC) patients and was positively associated with tumor grading. However, the function and underlying mechanisms of ANP32A in CRC metastasis have not been fully explored. In this study, we found that ANP32A knockdown significantly attenuated the migration and invasion, and epithelial-mesenchymal transition (EMT) in cells. Further mechanistic studies revealed that ANP32A knockdown inhibited the expression of β-catenin and phosphorylated-ERK. The immunofluorescent staining experiment has revealed that ANP32A was expressed in the cell membrane, cytosol and nucleus, and its expression was positively associated with β-catenin expression levels. Moreover, the ability of cell migration and invasion was inhibited, the expression of E-cadherin was enhanced following ANP32A knockdown, and these affects were abolished by an ERK activator PMA, enhanced by an ERK inhibitor PD98059. Moreover, our animal experiment also demonstrated that silenced ANP32A inhibited CRC cell growth, multi-organ metastasis, ERK activation and EMT progression *in vivo*. Collectively, these findings demonstrated that ANP32A promotes CRC progression and that may be a promising target for the anti-metastasis treatment of CRC.

## Introduction

Colorectal cancer (CRC) is a common digestive tract cancer and exhibits the second highest rate of cancer-related death^1^. To date, there are nearly 1,200,000 new cases of CRC and more than 600,000 deaths as a result of the disease each year^2^-^4^. The high rates of mortality are mainly due to recurrence and metastases^3^, ^5^, and tumor metastases account for ~90% of the deaths in patients with CRC^6^. It has been reported that CRC metastasis is closely related with epithelial-mesenchymal transition (EMT)^7^, which is a common behavior happened in tumor metastasis^8^. However, the potential molecular mechanisms underlying CRC metastasis and EMT are yet to be fully elucidated. Therefore, further investigations into novel biomarkers and molecular mechanisms involved in CRC metastasis may help to find new therapeutic strategies for CRC metastasis.

ANP32A also known as protein phosphatase 32 (PP32), is a type of nuclear phosphoprotein that is overexpressed in numerous cancers, including CRC^9^-^11^. Our previous study showed that there was a notable association between ANP32A and the tumor differentiation of CRC^9^. Yang *et al* demonstrated that ANP32A was upregulated in primary acute myeloid leukemia (AML) cells and was required for the proliferation and survival of AML cells^10^. Moreover, Velmurugan *et al* found that high ANP32A expression in cancer tissues was correlated with the metastasis of lymph node and poor survival of the patients with oral squamous cell carcinoma^11^. Moreover, ANP32A expression was closely associated with the activation of ERK^12^, which plays a vital role in CRC invasion, migration and EMT^13^-^15^. While the specific role of ANP32A and its underlying mechanism in CRC metastasis and EMT are yet to be fully elucidated. So, in this study, we explored the potential role of ANP32A in the treatment of metastasis in patients with CRC.

## Materials and methods

### Cell lines and culture

HCT116 cell line was obtained from the Academia Sinica Cell Bank. SW480 cell line was preserved in our laboratory. All culture medium used was DMEM supplemented with 10% FBS (Clark Bioscience) and 1% penicillin-streptomycin (Solarbio, china). Cells were kept in a 37°C and 5% CO_2_ incubator.

### Bioinformatics analysis

The gene expression profiling of ANP32A and β-catenin in CRC, and the correlation between the two genes were analyzed using an online database (https://www.xiantao.love/).

### ANP32A knockdown in CRC cells

SW480 and HCT116 cells were transfected with ANP32A short-hairpin RNA (shRNA) plasmid GV248 (hU6-MCS-Ubiquitin-EGFP-IRES-puromycin) for knockdown of ANP32A, and the sequences were as follows: 5'AAGCTTGAACTAAGCGATA3' (sh-ANP32A#1) and 5'TATTGTGATTTGACTGTTT3' (sh-ANP32A#2). The negative control (NC) plasmid GV248 was used as the control and the corresponding sequence was as follows: 5'TTCTCCGAACGTGTCACGT3'. SW480 and HCT116 cells were transfected with Lipofectamine®3000 for 2 days. CRC cells stably transfected with sh-ANP32A were selected following growth in puromycin for at least 3-6 days. Knockdown of ANP32A was detected by fluorescence microscopy, real-time quantitative (RT-q) PCR and western blot analysis.

### Cell proliferation assay

MTT assay was performed to analyze the cell viability. HCT 116 cells were seeded in 96-well plates, after the cells were treated with PD98059 or PMA at 24 h, 48 h and 72 h, 20 µL MTT (5 mg/mL) was added in each well of the 96-well plates detected. Then, the cell medium was discarded, and 150 µLDMSO was added to each well. Cell viability was evaluated using the 490 nm value of each well. PD98059 (MedChemExpress, CAS#167869-21-8) and phorbol myristate acetate (PMA; Good Laboratory Practice Bioscience, CAS#16561-29-8). PMA (0.05, 0.1, 0.2, 0.5 and 1μM) and PD98059 μM (1.25, 2.5, 5 and 10 μM) were used to select the appropriate concentration by the MTT assay.

### Transwell assay

Transfected SW480 cells (1x10^5^ in 200 µL/well) and HCT116 cells (3x10^5^ in 200 µL/well) were seeded into the Transwell chamber. Prior to cell seeding, the upper chambers were coated with basement membrane Matrigel and kept in the incubator for 3 h. Subsequently, the lower chamber was added with DMEM cell culture containing 15% FBS (600 µL/well). Then 1 μM PMA or 2.5 μM PD98059 was added to the chamber. Following 48 h treatment, cells under the upper chambers were washed with PBS and fixed with 4% paraformaldehyde. And then, the cells were stained with 0.1% crystal violet solution at 37℃ for 10mins. Migrated cells were observed and counted under a microscope.

### Wound healing assay

A total of 8x10^5^ (each well) CRC cells of the control (sh-NC) or sh-ANP32A were seeded in 6-well plates. Following cell seeding for 12 h, cells wound were caused by a 200-μl sterile pipette tip. After the culture medium changed, the cells were treated with 1 μM PMA or 2.5 μM PD98059. The migration of cells was photographed with a microscope at 0 and 48 h. The wound in each well was analyzed by ImageJ software (ImageJ 1.8.0.112).

### Immunofluorescent staining

CRC cells were seeded onto sterilized coverslips and incubated at 37 ℃ at least 24 h. After the slides were fixed, they were permeabilized with 0.1% Triton X-100 for 10mins. The slides were subsequently blocked with 5% bovine serum albumin (BSA; dissolved in PBS) for 1 h and incubated with β-catenin or ANP32A for at least 5 h at 4℃. Following washing with PBS four times for 30 mins, the fluorescent-labelled secondary antibody was added to the wells and then incubated for 1 h at 30℃. After washed with deionized water 3 times, cells were stained with DAPI (dissolved with deionized water) for 5 min in the dark. The cell staining was viewed and photographed under a confocal laser-scanning microscope (Olympus FV3000; Olympus Corporation).

### Xenograft mouse model

BALB/c nude mice (male, age 4-6 weeks) were obtained from Hunan SJA Experimental Animal Co., Ltd. Mice were randomly divided into sh-NC and sh-ANP32A groups, and there were 6 mice in each group. Stable HCT116 sh-NC and HCT116 sh-ANP32A cells (6x10^6^ cells/mice) were injected into the nude mice under the skin. 21 days later, mice were sacrificed using pentobarbital sodium, and all the tumors were removed, weighed and sectioned. To observe the function of ANP32A on CRC metastasis* in vivo*, a total of 5x10^6^ HCT116 cells/200 µL/mouse (sh-ANP32A or sh-NC) were intravenously injected into nude mice via their tails. The mice were divided into sh-NC and sh-ANP32A groups. The mice were sacrificed after eight weeks. The lungs, livers, heart, brain, spleen and kidneys of the mice were removed and fixed with phosphate-buffered formalin. Visible tumor metastatic nodules of each organ were calculated. All animal experimental procedures carried out in the present study were approved by the Animal Ethics Committee of Guilin Medical University (approval no. GLMC-IACUC-2021011) and conducted in accordance with the Guideline of the Care and Use of Laboratory Animals in Guilin Medical University.

### RNA extraction and RT-qPCR

Total RNA of the CRC cells was extracted using TRIzol (Tiangen). A Revert Aid First Strand cDNA Synthesis kit was employed to reverse transcribed the extracted RNA. And the gene expression was quantitatively assessed using ABI PowerUp™ SYBR™ Green Master Mix and the AB1 7500fast Real-Time Detection System. The primer sequences of ANP32A and β-actin (Invitrogen; Thermo Fisher Scientific, Inc.) were the same as previously reported^9^. Fold changes of ANP32A were calculated using the 2^-ΔΔCt^ method.

### Western blot analysis

Total protein of HCT116 or SW480 cells was extracted by protein lysis buffer. BCA kit (Beyotime, China) was used to measure the cells protein concentration. Tissue lysate (30 µg) was separated by 10-12% SDS-PAGE electrophoresis and transferred to nitrocellulose membranes. After the membranes blocked, it was incubated with primary antibodies, including anti-ANP32A (Abcam Cat. No: ab5992), anti-E-cadherin (Cell Signaling Technology 3195T), anti-N-cadherin (Wanleibio WL01047), anti-β-catenin (Abcam Cat. No: ab32572), anti-Akt (Abcam Cat. No: ab179463), anti-phosphorylated (p)-Akt (14C10), (Wanleibio WLP001a), anti-Vimentin (Abcam Cat. No: ab92547), anti-ERK (Abcam Cat. No: ab184699), anti-p-ERK (Thr202/Tyr204) (Wanleibio WLP1512) and anti-β-actin (ZSGB-BIO Cat. No: TA-09). After that, the secondary HRP-linked antibody was used to incubate with the membranes. Protein bands were analyzed using the chemiluminescence detection system.

### Statistical analyses

SPSS 21.0 software was used to perform the statistical analysis of this study. Paired Student's t-test or one-way ANOVA followed by Bonferroni's post hoc test were used to analyze our data in this study. Data are presented as the mean ± SD, *P*<0.05 was considered statistically significant*.*

## Results

### ANP32A knockdown inhibits CRC cell migration and invasion

**Figure 1:**
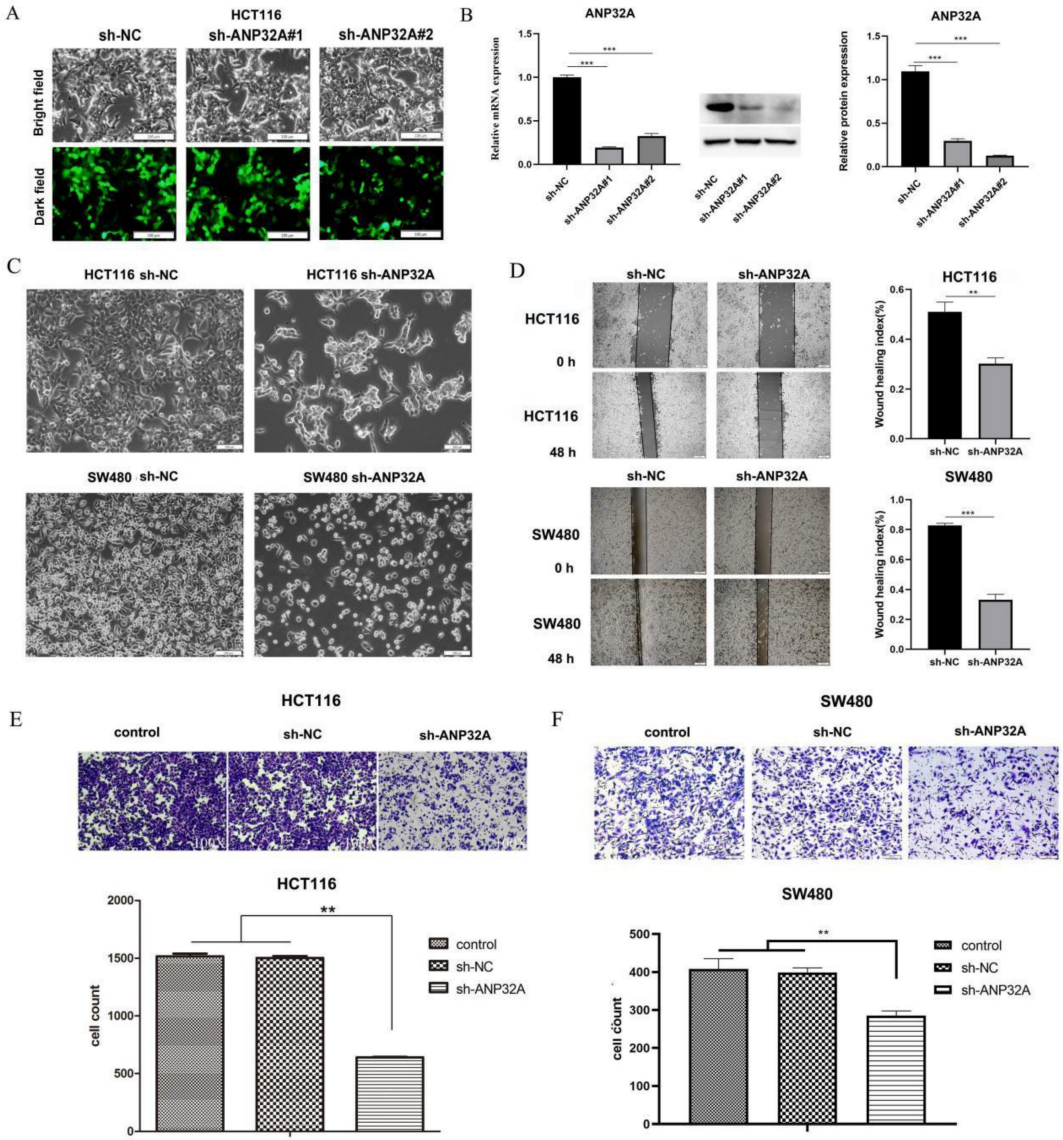
** ANP32A knockdown inhibits CRC cell migration and invasion.** (A and B) ANP32A knockdown was carried out using two sh-ANP32A plasmids, and successful knockdown was determined using fluorescence microscopy, reverse transcription-quantitative PCR, and western blot analyses. (C) Morphological changes of HCT116 and SW480 cells were observed using an inverted phase contrast microscope. (D) The migration of sh-ANP32A cells was analyzed using a wound healing assay. (E and F) The invasion of sh-ANP32A cells was detected using a Transwell assay. All data are presented as the mean ± standard deviation and n= 3. ^**^P <0.01, ^***^P<0.001 vs. sh-NC. ANP32A, acidic leucine rich nuclear phosphoprotein-32A; CRC, colorectal cancer; sh, short hairpin RNA; NC, negative control.

To explore the role of ANP32A in CRC metastasis, HCT116 cells were transfected with the GV248 plasmid for ANP32A knockdown. As shown in Fig. 1A and B, the expression of ANP32A was reduced following transfected with sh-ANP32A#1 and sh-ANP32A#2 two plasmids. However, the highest reduction was observed following transfection with sh-ANP32A#2. Thus, the sh-ANP32A#2 plasmid was selected for subsequent studies. Following ANP32A knockdown, the morphology of SW480 and HCT116 cells was altered compared with NC cells. Our above results also demonstrated that the expression of ANP32A inhibited led to increased cell-cell contact and a reduced stretched/elongated spindle-like morphology, particularly in HCT116 cells (Fig. 1C) and SW620 (Fig. S1). A stretched/elongated spindle-like morphology and a reduction in cell-cell contact is indicative of an aggressive phenotype^16^.

To observe the effects of ANP32A knockdown on CRC cell migration and invasion, we performed wound healing and Transwell assays. As Fig. 1D shown, ANP32A knockdown in SW480 and HCT116 cells significantly reduced cell migration. Similar results were obtained from the Transwell assay, in which ANP32A knockdown markedly reduced cell invasion (Fig. 1E and F). These results indicated that ANP32A facilitated CRC cells' migration and invasion.

### ANP32A knockdown inhibits EMT in CRC cells

**Figure 2:**
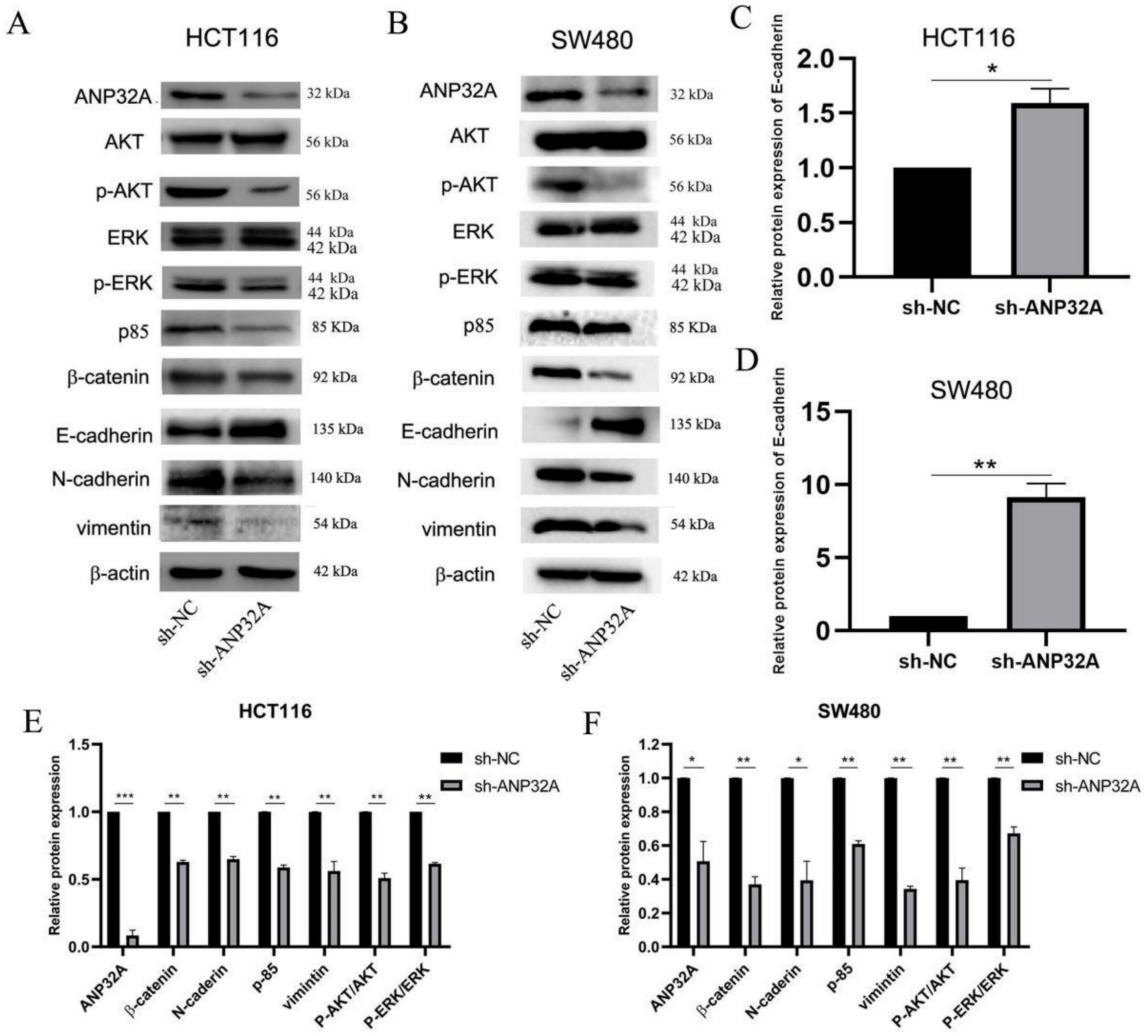
** ANP32A knockdown affects the expression of proteins associated with EMT via the ERK and Akt pathway in CRC cells.** (A) Representative western blotting image of EMT-, ERK- and Akt-associated proteins expressed in ANP32A knockdown HCT116 cells. (B) Representative western blotting image of EMT- and ERK-associated proteins expressed in ANP32A knockdown SW480 cells. (C-F) Quantification of the western blotting bands in ANP32A knockdown CRC cells. All data are presented as the mean ± standard deviation and n=3. ^*^P<0.5, ^**^P<0.01 vs. sh-NC. ANP32A, acidic leucine rich nuclear phosphoprotein-32A; EMT, epithelial-mesenchymal transition; CRC, colorectal cancer; sh, short hairpin RNA; NC, negative control.

One of the classical features of EMT is that the cells were changed into a stretched/elongated spindle-like morphology^17^. The ability of cell migration and invasion enhanced is closely associated with EMT^17^. ANP32A knockdown led to HCT116 cells and SW480 cells cell-cell contact increased and a reduced stretched/elongated spindle-like morphology, which indicated a decrease in the migration and invasion ability of CRC cells(as Fig. 1C shown). So, we speculated that ANP32A may promote the process of EMT. It has been demonstrated that the EMT-like morphologic and phenotypic changes are associated with the inhibition of E-cadherin expression and the enhancement of Vimentin, N-cadherin and β-catenin expression^18^. So, we detected these factors' expressions in ANP32A knockdown CRC cells. As Fig. 2 A-F shown, the protein level of E-cadherin was obviously increased in the sh-ANP32A group compared with the sh-NC group, whereas the expression of β-catenin, Vimentin and N-cadherin were significantly decreased.

### Location and association between ANP32A and β-catenin in CRC

**Figure 3:**
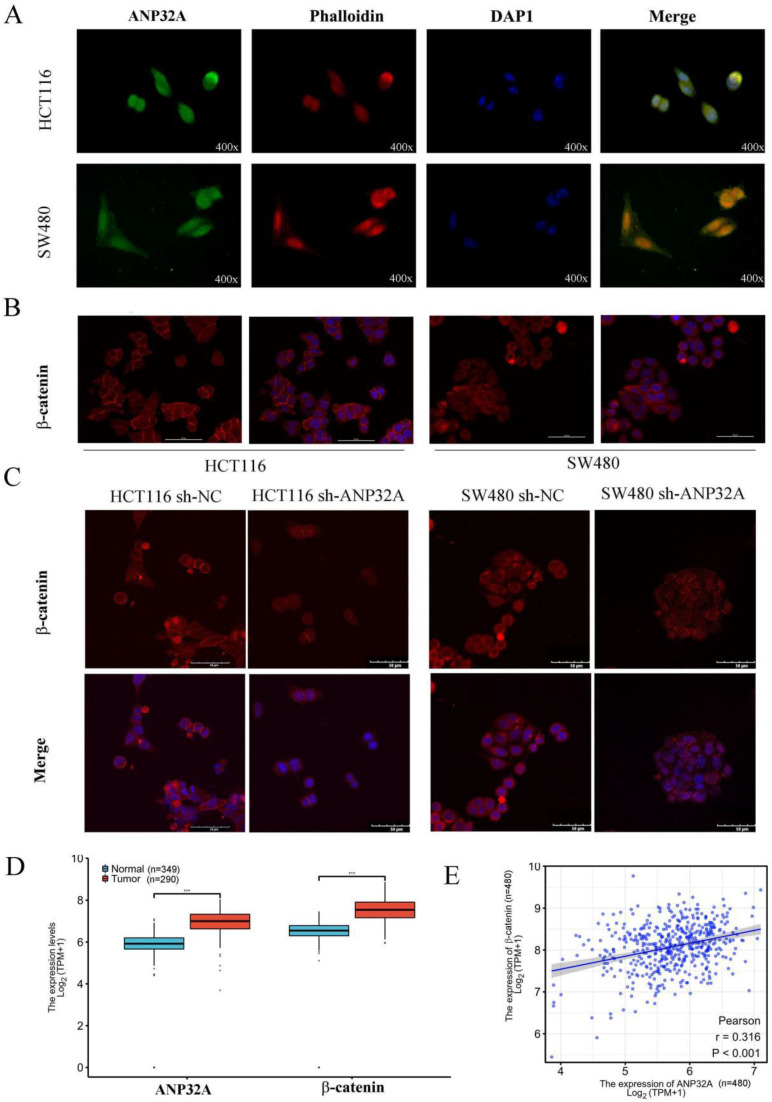
**Location and correlation between ANP32A and β-catenin in CRC cells.** (A) Expression and cellular localization of ANP32A (magnification, x400). (B) Expression and cellular localization of β-catenin in CRC cells (scale bar, 50 µm). (C) The fluorescent density of β-catenin was measured in ANP32A knockdown CRC cells (scale bar, 50 µm). (D) Results of the online database demonstrated that ANP32A and β-catenin gene expression levels were significantly upregulated in CRC (n=290) compared with normal tissues (n=349). (C) The correlation between ANP32A and β-catenin was analyzed in CRC samples (n=480). ANP32A, acidic leucine rich nuclear phosphoprotein-32A; CRC, colorectal cancer.

The cellular location of certain factors was closely associated with cell metastasis; thus, the cellular location of ANP32A and β-catenin were analyzed using immunofluorescence. As Fig. 3A and Fig. 3B shown, ANP32A was expressed in the cell membrane, cytosol and nucleus, and β-catenin was mainly located in the cell membrane. Consistent with the western blotting results of this study, ANP32A knockdown decreased the expression of whole cell β-catenin, including β-catenin localized to the cell membrane (Fig. 3C). Subsequently, the expression profiling of ANP32A and β-catenin in CRC patients were analyzed using the TCGA-GETx database, and we found that the levels of ANP32A and β-catenin were obviously higher in 290 COAD tissues compared with 349 healthy tissues (Fig. 3D). Moreover, As the Pearson's correlation analysis shown (Fig. 3E), the correlation between β-catenin and ANP32A was 0.316 in 480 samples (P<0.001).

### ANP32A knockdown inhibits CRC cell migration and invasion by suppressing ERK activation

**Figure 4:**
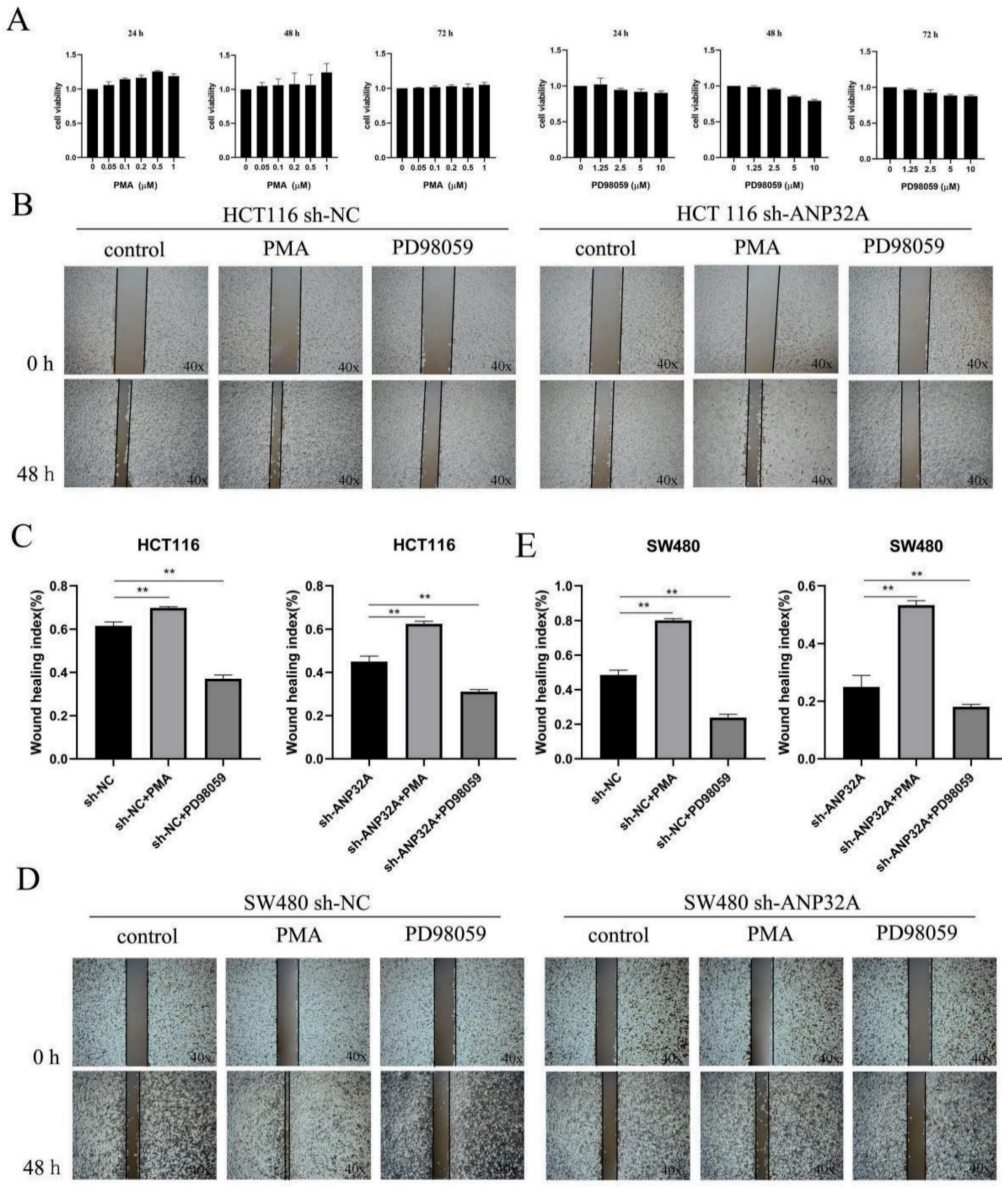
** Cell migration is altered in ANP32A knockdown CRC cells following treatment with PMA and PD98059.** (A) Cell viability was detected in SW480 cells following treatment with PMA and PD98059. (B and C) Representative image and quantification of HCT116 cells. (D and E) Representative image and quantification of the SW480 cells. All data are presented as the mean ± standard deviation and n=3. ^**^P<0.01 vs. sh-NC. ANP32A, acidic leucine rich nuclear phosphoprotein-32A; CRC, colorectal cancer; sh, short hairpin RNA; NC, negative control; PMA, phorbol myristate acetate.

**Figure 5:**
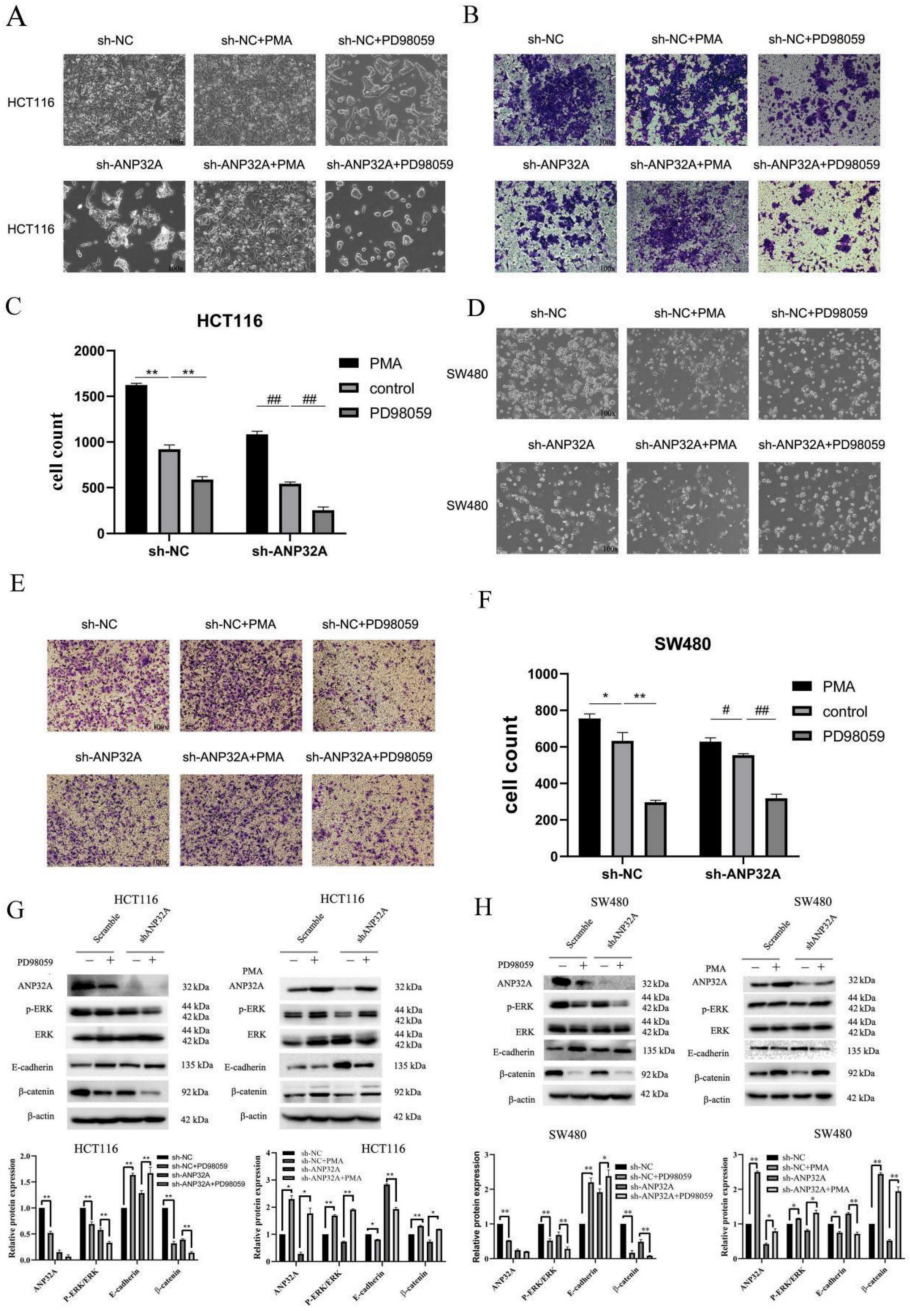
** Cell invasion is altered in ANP32A knockdown CRC cells following treatment with PD98059 and PMA.** (A and D) Morphological changes in CRC cells were observed following treatment with PMA and PD98059. (B, C, E and F) Invasion of sh-ANP32A cells was detected using a Transwell assay (magnification, x100). All data are presented as the mean ± standard deviation and n=3. ^*^P<0.5, ^**^P<0.01 vs. sh-NC; ^#^P<0.5, ^##^P<0.01 vs. sh-ANP32A. (G) Expression levels of E-cadherin, β-catenin and p-ERK were detected using western blotting in ANP32A knockdown HCT116 cells. (H) Expression levels of E-cadherin, β-catenin and p-ERK were detected using western blotting in ANP32A knockdown SW480 cells. All data are presented as the mean ± standard deviation and n= 3. ^*^P<0.5, ^**^P<0.01 vs. sh-NC. ANP32A, acidic leucine rich nuclear phosphoprotein-32A; CRC, colorectal cancer; sh, short hairpin RNA; NC, negative control; p-, phosphorylated; PMA, phorbol myristate acetate.

The activation of MEK/ERK pathway is an important factor for EMT, invasion and migration in cancer cells. Our above results showed that ERK activation was inhibited in sh-ANP32A HCT116 and SW480 cells (Fig. 2A-F). As demonstrated in Fig. 1C, sh-ANP32A promoted a morphological change to a less aggressive phenotype (decreased elongation and increased cell-cell contact). Moreover, ANP32A knockdown suppressed ERK activation. Thus, the impact of ERK on the metastasis of CRC cells, and its effects on the expression of ANP32A were assessed following treatment with PD98059 (an inhibitor of ERK) and phorbol myristate acetate (PMA; an inducer of ERK activation). In total, 1 μM PMA and 2.5 μM PD98059 were selected for use in further experiments, following a cell viability assay (Fig. 4A). As Fig. 4B-E shown, the reduction in the migration of CRC cells in response to ANP32A knockdown was reversed following treatment with PMA, and the inhibition effect was enhanced following treatment with PD98059. Moreover, PD98059 treatment enhanced the effects of ANP32A knockdown on the morphological changes in CRC cells, while PMA induced a more aggressive phenotype in CRC cells, which was inhibited following ANP32A knockdown (Fig. 5A and D). Consistent with the observed changes in cell morphology, PMA treatment significantly increased CRC cell invasion and reduced the inhibitory effects of ANP32A knockdown on cell invasion. By contrast, PD98059 treatment caused the opposite phenotype (Fig. 5A-F). Furthermore, knockdown of ANP32A obviously inhibited phosphorylation levels of ERK and the expression of β-catenin, which were significantly enhanced following treatment with PD98059 and reduced following treatment with PMA in HCT116 and SW480 cells. Moreover, as Fig. 5G and H shown, the altered expression of E-cadherin induced by ANP32A knockdown was enhanced following treatment with PD98059 and abolished following treatment with PMA. The above-mentioned results indicated that the stimulatory effects of ANP32A on cell migration, invasion and EMT progress in CRC cells are associated with ERK activation.

### ANP32A knockdown inhibits CRC aggressiveness in nude mice

**Figure 6:**
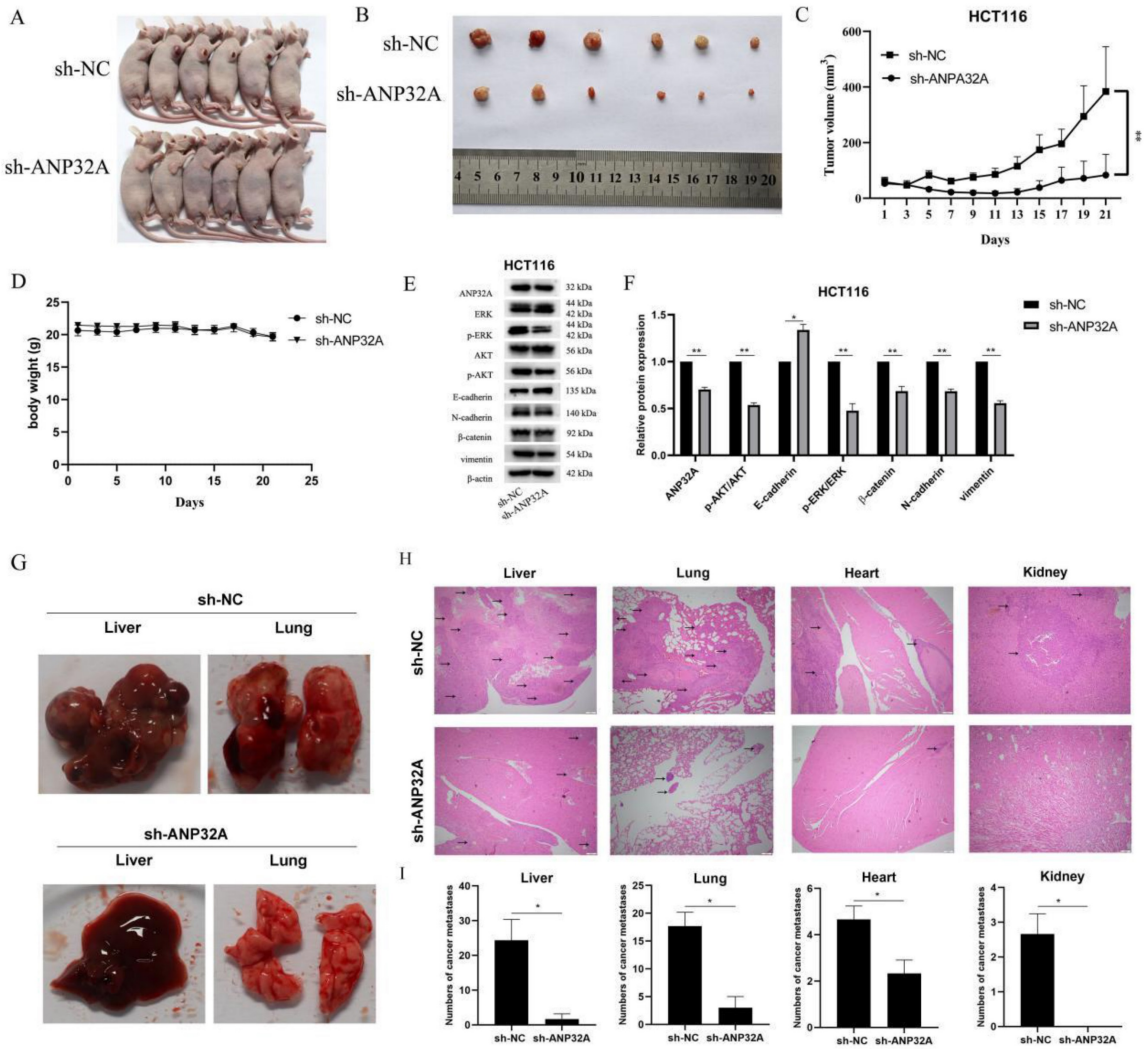
** ANP32A knockdown inhibits CRC aggressiveness in nude mice.** (A and B) ANP32A knockdown markedly reduced tumor growth *in vivo*. (C)Tumor volume was recorded every 2 days. (D)The body weight of nude mice was recorded every 2 days. (E) Expression of proteins were determined using western blotting. (F) The intensity of bands was quantified using Image Lab software, and β-actin was used as the internal control. (G) A representative diagram of the liver and lung metastasis in the metastatic nude mouse model. (H) A representative image of the liver, lung, heart, and kidney of the metastatic nude mouse model following H&E staining. (I) Statistical analysis of the liver, lung, heart and kidney metastases in the nude mouse model. Black arrows represent metastases. All data are presented as the mean ± standard deviation. ^**^P<0.01, ^**^P<0.001 vs. sh-NC. ANP32A, acidic leucine rich nuclear phosphoprotein-32A; CRC, colorectal cancer.

To investigate the effect of ANP32A on CRC cells *in vivo*, a tumor xenograft model was established by the HCT116 cells transfected with sh-ANP32A or sh-NC. As Fig.6A-C shown, ANP32A knockdown significantly reduced tumor volume *in vivo*, and there was no notable mice body weight between the sh-NC and sh-ANP32A groups (Fig. 6D). ANP32A knockdown was further verified using western blot assay (Fig. 6E). Our western blot results demonstrated that the expression of Vimentin, β-catenin and N-cadherin (EMT-related markers) were markedly reduced in the ANP32A knockdown group, while the expression of E-cadherin was increased. Moreover, the activation of ERK was also inhibited in ANP32A knockdown tumor cells (Fig. 6E and F). The functional impact of ANP32A on metastasi*s* was investigated* in vivo* using a nude mouse metastatic tumor model. Following 8 weeks, loss of body weight occurred in the untreated group. Following pathological dissection, visual observation (Fig. 6G), H&E staining (Fig. 6H) and statistical analysis (Fig. 6I) demonstrated that sh-NC tumor cells exhibited multiple organ metastasis to the liver, lung, heart and kidney. Notably, the liver possessed the most metastatic nodules compared with all other metastasis organs. Compared with the sh-NC mice, the ANP32A knockdown mice showed obviously lower levels of tumor metastasis, a lower number of involved organs and a reduction in metastases. Collectively, these data revealed the role of ANP32A in the EMT and metastasis of CRC, and its promotion of metastatic ability is closely associated with ERK activation.

## Discussion

ANP32A is a multifunctional protein that participates in cell apoptosis, tumorigenesis, and neurodegenerative diseases. Previous studies revealed that ANP32A showed tumor inhibition effect on prostate cancer^19^, breast cancer^20^ and pancreatic cancer cells^21^. Moreover, recently multiple studies found that ANP32A may function as a tumor promotor^9^, ^11^, ^12^. Our previous study demonstrated that the ANP32A was highly expressed in CRC tissues, and subsequent knockdown using small interfering RNA may inhibit CRC cell proliferation^9^. Velmurugan *et al* found that ANP32A knockdown decreased the invasion and metastasis of squamous cell carcinoma HSC-3 cells^11^. However, the specific function and underlying mechanisms of ANP32A in the progression of CRC are still unclearly.

In this study, ANP32A knockdown inhibited SW480 and HCT116 cell invasion, migration, and metastasis. EMT promotes colorectal cancer invasion and metastasis^22^, and is closely associated with malignant tumor recurrence^23^. During the process of EMT, the protein expression of E-cadherin is decreased, while the expression of N-cadherin and Vimentin are increased in cancer cells^2^. In the present study, the expression of Vimentin and N-cadherin were reduced, while the expression of E-cadherin was elevated in sh-ANP32A cells. Thus, ANP32A knockdown caused EMT inhibition, and suppressed the migration and invasion of CRC cells. Moreover, our results also demonstrated that HCT116 cells predominantly metastasize to the liver, and then to the lung, which is consistent with a previous report^24^, ^25^. ANP32A knockdown reduced the number of metastatic organs and metastatic nodules. These results also verified that ANP32A knockdown inhibited the ability of CRC cells to metastasize *in vivo.*

Numerous previous studies reported that the MAPK/ERK pathway is pivotal for the development of CRC^3^, ^26^, ^27^. C-X-C motif chemokine ligand 5(CXCL5) enhanced the cell migration and invasion ability of CRC by inducing EMT through the activation of ERK^28^. Results of a previous study demonstrated that MAPK/ERK activated by Forkhead box protein C2(FOXC2) induced CRC EMT and promoted oxaliplatin resistance^27^. Moreover, sphingolipid transporter 2 (SPNS2) promoted CRC cell migration and invasion via ERK activation^29^. Results of our previous study demonstrated that ANP32A promoted CRC cell proliferation through p38 inhibition and Akt signaling pathway activation^9^. Sun *et al* demonstrated that ANP32A was critical for the maintenance of a hyper-proliferative and undifferentiated status in Acute megakaryoblastic leukemia (AMKL) cells, and ANP32A contributed to the pathogenesis of AMKL. Moreover, ANP32A reduced the induction of RUNT-related transcription factor 1 (RUNX1) and Friend leukemia integration 1 (FLI1), and inhibited ERK activation induced by PMA^12^. Therefore, determining the specific role of ANP32A in the MAPK/ERK pathway may uncover the molecular mechanism underlying ANP32A in CRC metastasis. In this study, ANP32A knockdown decreased the expression of p-ERK. In addition, the inhibition of β-catenin, a key activator of EMT^30^, caused by ANP32A knockdown was abrogated following treatment with ERK activator PMA, and enhanced following treatment with ERK inhibitor PD98059. Yang *et al* demonstrated that ERK phosphorylation induced by IL-17RB increased β-catenin expression and promoted lung cancer metastasis 31. ERK is an vital upstream regulator of the β-catenin signaling pathway^32^, its activity may be suppressed following treatment with antitumor drugs or the downregulation of oncogenes^33^-^35^. In the current study, the expression of β-catenin was highly expressed in patients with ANP32A-high CRC. Moreover, there was a decrease in β-catenin expression and ERK activation following the ANP32A knockdown. This indicates that ANP32A plays a a role in promoting CRC EMT and metastasis through the upregulation of the ERK/β-catenin signaling pathway. However, further studies are required to dissect how ANP32A induces morphological changes in CRC cells, and to understand the specific mechanistic actions between ANP32A, ERK and β-catenin in CRC progression, particularly during metastasis.

In conclusion, this demonstrated that ANP32A functioned as a promoter of CRC metastasis and EMT progression by influencing the phosphorylation of ERK. These results revealed that ANP32A may act as a target of anti-metastasis for the treatment of CRC, and further demonstrated the potential mechanisms underlying ANP32A silencing in antimetastatic treatment for human CRC. Notably, there are some limitations in this study, including the small number of mice included, and a lack of investigation into the association between ANP32A and Akt, which will be the direction of future investigations.

## Supplementary Material

Supplementary figure.
