# Supplementary figures and images for: ANP32A Knockdown Attenuates the Malignant Biological Behavior of Colorectal Cancer Cells by Suppressing Epithelial-mesenchymal Transition and ERK Activation
Source: J Cancer. 2023 Sep 4;14(15):2759–70. doi: 10.7150/jca.84687 (PMC10539559; doi:10.7150/jca.84687)

SW620

sh-NC

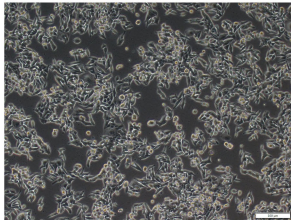

sh-ANP32A

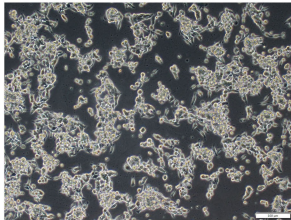

Supplement: Supplementary file 1 — Supplementary figure. [file jcav14p2759s1.pdf]
